# Uncovering the potential molecular mechanism of liraglutide to alleviate the effects of high glucose on myoblasts based on high-throughput transcriptome sequencing technique

**DOI:** 10.1186/s12864-024-10076-w

**Published:** 2024-02-08

**Authors:** Dongmei Fan, Yunjie Zhang, Lanyu Lu, Fuzai Yin, Bowei Liu

**Affiliations:** 1 Department of Endocrinology, The First Hospital of QinHuangdao, 258 Wenhua Road, Haigang District, Qinhuangdao City, 066000 Hebei Province China; 2 Department of Nursing, The First Hospital of QinHuangdao, Qinhuangdao City, 066000 Hebei Province China

**Keywords:** Liraglutide, C2C12, Myoblasts, High glucose, Metabolism, DE-mRNAs

## Abstract

**Background:**

Myoblasts play an important role in muscle growth and repair, but the high glucose environment severely affects their function. The purpose of this study is to explore the potential molecular mechanism of liraglutide in alleviating the effects of high glucose environments on myoblasts.

**Methods:**

MTT, western blot, and ELISA methods were used to investigate the role of liraglutide on C2C12 myoblasts induced by high glucose. The high-throughput transcriptome sequencing technique was used to sequence C2C12 myoblasts from different treated groups. The DESeq2 package was used to identify differentially expressed-mRNAs (DE-mRNAs). Then, functional annotations and alternative splicing (AS) were performed. The Cytoscape-CytoHubba plug-in was used to identify multicentric DE-mRNAs.

**Results:**

The MTT assay results showed that liraglutide can alleviate the decrease of myoblasts viability caused by high glucose. Western blot and ELISA tests showed that liraglutide can promote the expression of AMPKα and inhibit the expression of MAFbx, MuRF1 and 3-MH in myoblasts. A total of 15 multicentric DE-mRNAs were identified based on the Cytoscape-CytoHubba plug-in. Among them, Top2a had A3SS type AS. Functional annotation identifies multiple signaling pathways such as metabolic pathways, cytokine-cytokine receptor interaction, cAMP signaling pathway and cell cycle.

**Conclusion:**

Liraglutide can alleviate the decrease of cell viability and degradation of muscle protein caused by high glucose, and improves cell metabolism and mitochondrial activity. The molecular mechanism of liraglutide to alleviate the effect of high glucose on myoblasts is complex. This study provides a theoretical basis for the clinical effectiveness of liraglutide in the treatment of skeletal muscle lesions in diabetes.

**Supplementary Information:**

The online version contains supplementary material available at 10.1186/s12864-024-10076-w.

## Introduction

Myoblasts are stationary skeletal muscle satellite cells with the function of muscle growth and repair [1]. Myoblasts fuse to form myotubes involved in regeneration and repair of damaged/injured skeletal muscle [2]. Myoblasts transplantation is also a potential treatment for urological dysfunction and heart failure [3]. The adverse effects of high glucose concentration on cells are called glucose toxicity. High ambient glucose inhibits the myogenesis of C2C12 myoblasts, characterized by a two-fold decrease in myoblasts fusion, a decrease in intracellular of myogenic determination factor (MyoD), myogenic and myosin heavy chain (MHC) levels, and increased cellular content of active myostatin isoform [4]. High glucose stress also affected myoblasts survival and mitochondrial function [5]. A previous study found that hyperglycemia negatively affects induced pluripotent stem cells (IPSC)-derived myoblasts proliferation, skeletal muscle regeneration and function. Moreover, in hyperglycemic myoblasts, cell cycle progression is blocked in S phase and G2/M phase, and mitochondrial function is impaired [6]. Therefore, exploring how to inhibit the adverse effects of high glucose on myoblasts is helpful for the management and treatment of skeletal muscle lesions in diabetes.

Liraglutide is an acylated glucagon-like peptide-1 (GLP-1) analogue, which has 97% amino acid homology with natural GLP-1 and has significant long-term effect [7]. Liraglutide has protective effects on freezing injury, denervation, and dexamethasone induced skeletal muscle atrophy and improved muscular function [8]. Liraglutide can also affect insulin resistance in skeletal muscle cells [9]. Liraglutide prevents cardiac systolic function impairment, reactive oxygen species (ROS)/O^2−^ production, cell apoptosis, and mitochondrial damage induced by short-term high glucose incubation [10]. Liraglutide can prevent high glucose induced dysfunction of human umbilical vein endothelial cells (HUVECs) by inhibiting PTEN-induced putative kinase 1 (PINK1)/Parkin dependent mitophagy [11]. Liraglutide also protects nucleus pulposus cells and fibroblasts stimulated via high glucose by activating related pathways [12, 13]. So far, it has not been found whether liraglutide protects myoblasts stimulated by high glucose.

**Fig. 1:**
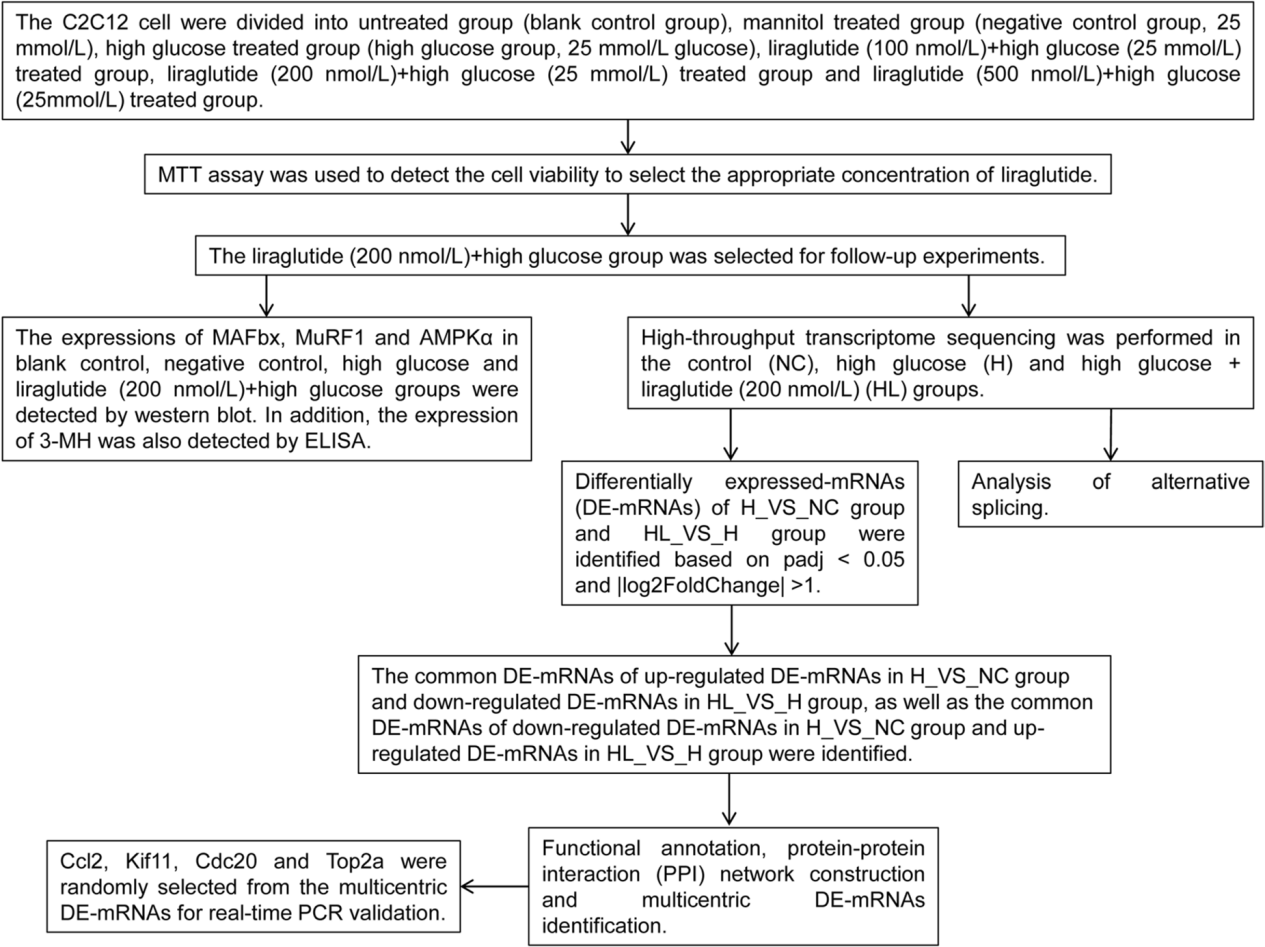
The flowchart of the study

Muscle atrophy F-box (MAFbx) and muscle RING finger 1 (MuRF1) are considered to be key regulators of muscle protein degradation [14, 15]. AMP-activated protein kinase (AMPK) can detect intracellular energy levels and is responsible for regulating the cell's energy supply system to maintain cell metabolism. It has specific regulation of various aspects of mitochondrial biology and homeostasis [16]. It not only acts as an energy switch that controlling anabolism and catabolism, but also as a sensor for glucose and cellular energy status [16, 17]. 3-methylhistidine (3-MH) is a byproduct of actin and myosin degradation [18]. In this study, the expression of MAFbx, MuRF1 and AMPKα and 3-MH in each group were verified by WB and ELISA, respectively, to detect the alleviating effect of liraglutide on high glucose induced myoblasts. In addition, high-throughput transcriptome sequencing technique was also used to explore the molecular mechanism of liraglutide affecting C2C12 myoblasts stimulated by high glucose (Fig. 1). This study provides a theoretical basis for the clinical effectiveness of liraglutide in the treatment of skeletal muscle lesions in diabetes.

## Materials and methods

### In vitro validation of the effect of liraglutide on myoblast under high glucose condition

C2C12 cells were used for experimental research in vitro. C2C12 cells line (item number: CL-0044) was purchased from Procell Life Science& Technology Co., Ltd. The C2C12 cells were divided into untreated group (blank control group), mannitol treated group (negative control group, 25 mmol/L), high glucose treated group (high glucose group, 25 mmol/L glucose), liraglutide (100 nmol/L) + high glucose (25 mmol/L) treated group, liraglutide (200 nmol/L) + high glucose (25 mmol/L) treated group and liraglutide (500 nmol/L) + high glucose (25 mmol/L) treated group. The treatment time of each group was 48 h. The 3-(4,5-dimethylthiazol-2-yl)-2,5-diphenyl-2H-tetrazolium bromide (MTT) assay was used to detect the cell viability to select the appropriate concentration of liraglutide. Optical density (OD) value was measured at 490 nm wavelength. The expressions of MAFbx, MuRF1 and AMPKα in each group were detected by western blot. The names of the primary antibodies used were AMPKα antibody (A1229, Abclonal), Fbx32 antibody (A3193, Abclonal), TRIM63 antibody (A3101, Abclonal) and β-actin antibody (EM21002, Huaan biology). The name of the secondary antibody used was goat anti-rabbit IgG/HRP. The ELISA kit was used to detect the content of 3-MH in each group. OD value was measured at 450 nm wavelength.

### mRNA library construction, sequencing and raw data processing

The sequencing samples in this study included C2C12 cells control group (NC group), C2C12 cells high glucose (25 mmol/L) treated group (H group) and C2C12 cells high glucose (25 mmol/L) + liraglutide (200 nmol/L) treated group (HL group). Total RNA was extracted from cell samples using Trizol kit. Firstly, the mRNA was enriched and fragmented. Then, cDNA synthesis, terminal repair and connector linking were performed. Subsequently, polymerase chain reaction (PCR) reaction and product recovery were performed to complete the preparation of the library. Agilent 2100 BioAnalyzer was used to detect library quality. The Illumina HiSeq high-throughput sequencing platform was used to sequence cDNA library. Fastp was used for quality control of sequencing raw data. Specifically, adapter sequence, 5’ segment, 3’ segment, bases with quality < 20 and reads with *N* > 10% were trimmed. In addition, small fragments with length less than 25 bp after quality trimming were discarded. Hisat2 (version 2.1.0, https://ccb.jhu.edu/software/hisat2/index.shtml) was used to align the clean data after quality control to the human reference genome. Stringtie (version 1.3.3b, http://ccb.jhu.edu/software/stringtie/) calculates the fragments per kilobase of exon model per million mapped reads (FPKM) value of each gene / transcript in the sample according to the results in Hisat2, and used this value as the expression level of mRNA in the sample.

### Differential expression analysis of mRNA

The DESeq2 package was used to identify differentially expressed-mRNAs (DE-mRNAs) [19]. Differential expression analysis is mainly divided into three steps: (1) Standardize the original read count, mainly to correct the sequencing depth; (2) The hypothesis test probability (*P*-value) was calculated by statistical model; (3) Multiple hypothesis test correction (Benjiamini and Hochberg method) was performed to obtain padj value (also known as corrected P value or false discovery rate). The filter parameters were set to padj < 0.05 and |log2FoldChange (log2FC)|> 1.

### Functional annotation of common DE-mRNAs

Functional annotation was performed on the common DE-mRNAs of up-regulated DE-mRNAs in H_VS_NC group and down-regulated DE-mRNAs in HL_VS_H group, as well as the common DE-mRNAs of down-regulated DE-mRNAs in H_VS_NC group and up-regulated DE-mRNAs in HL_VS_H group. David database (https://david.ncifcrf.gov/tools.jsp) was used for Gene Ontology (GO) and Kyoto Encyclopedia of Genes and Genomes (KEGG) functional enrichment analysis of common DE-mRNAs. The screening criteria was *P*-value < 0.05.

### Identification of multicentric DE-mRNAs

In order to explore the interaction between common DE-mRNAs, a protein–protein interaction (PPI) network was constructed based on the STRING database (https://string-db.org/). Then, import the results obtained in the STRING database into Cytascape software (http://www.cytoscape.org/). Subsequently, maximum neighborhood component (MNC), degree, edge percolated component (EPC) and closeness in the Cytoscape-CytoHubba plug-in were used to screen multicentric DE-mRNAs.

### Analysis of alternative splicing (AS)

RMATS (http://rnaseq-mats.sourceforge.net/index.html) is a software developed for the analysis of AS for RNA-seq data. It can not only classify AS events, but also analyze the differences of AS events between different samples. The screening criteria for differentially AS events were false discovery rate (FDR) < 0.05 and |IncLevelDifference|> 0.05. RMATS divides the identified AS events into skipped exon (SE), alternative 5' splice site (A5SS), alternative 3' splice site (A3SS), mutually exclusion exon (MXE) and retained intron (RI).

### Real-time PCR validation

A total of 9 myoblasts samples were selected for real-time PCR verification, including 3 control samples, 3 H samples and 3 HL samples. Total RNA was extracted from cell samples using Trizol kit. FastQuant cDNA first strand synthesis kit and SuperReal PreMix Plus (SYBR Green) were used for reverse transcription and fluorescence quantitative detection, respectively. The data were quantitatively analyzed by 2^−△△CT^.

### Statistical analysis

Padj < 0.05 and |log2FC|> 1 were the screening criteria for DE-mRNAs. FDR < 0.05 and |IncLevelDifference|> 0.05 were the screening criteria for differentially AS. MTT, western blot and ELISA results were statistically analyzed using GraphPad Prism. One-way ANOVA test was used for statistical analysis. *P* < 0.05 was considered as statistical significance. This experiment was performed on cultured cell samples and no human samples were collected. Therefore, no sample sizes. Experiments were repeated independently at least 3 times. At present, the design of 3 biological repeated experiments is mainly used. The same experiment usually needs to be repeated at least 3 times to reduce experimental errors and avoid the impact of accidental phenomena on the experimental results. Data are presented as the mean ± standard deviation (SD).

## Results

### In vitro validation of the effect of liraglutide on C2C12 under high glucose condition

**Fig. 2:**
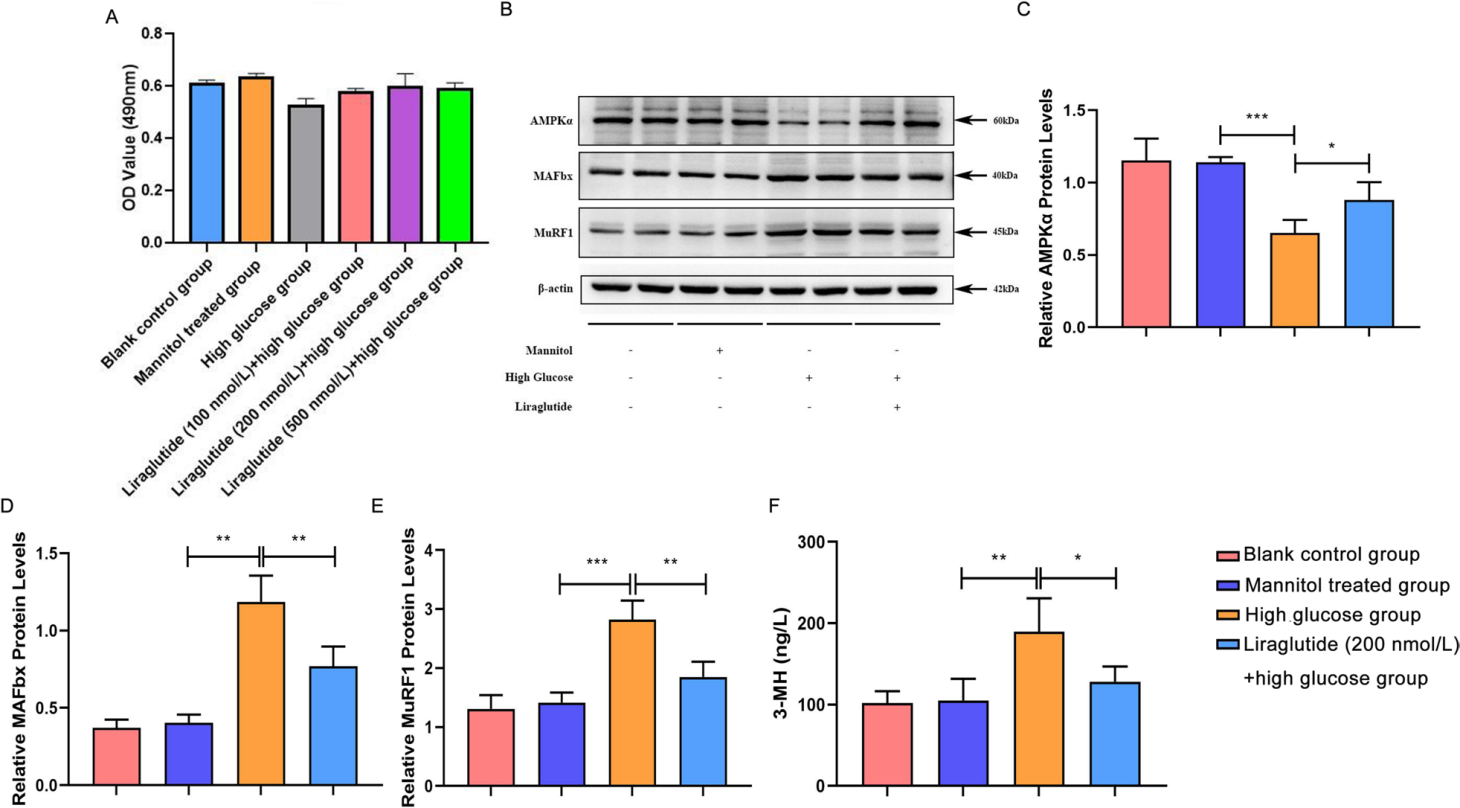
In vitro validation of the effect of liraglutide on C2C12 under high glucose condition. **A**: MTT was used to detect cell viability in blank control, mannitol treated, high glucose, liraglutide (100 nmol/L) + high glucose, liraglutide (200 nmol/L) + high glucose and liraglutide (500 nmol/L) + high glucose groups; **B**: Protein expression bands of AMPKα, MAFbx and MuRF1 in blank control, mannitol treated, high glucose and liraglutide (200 nmol/L) + high glucose groups. The blots were cropped and the full-length blots/gels are presented in Supplementary material-Original blot. **C**: Statistical histogram of AMPKα protein expression in blank control, mannitol treated, high glucose and liraglutide (200 nmol/L) + high glucose groups; **D**: Statistical histogram of MAFbx protein expression in blank control, mannitol treated, high glucose and liraglutide (200 nmol/L) + high glucose groups; **E**: Statistical histogram of MuRF1 protein expression in blank control, mannitol treated, high glucose and liraglutide (200 nmol/L) + high glucose groups; **F**: The content of 3-MH in the cell supernatant of blank control, mannitol treated, high glucose and liraglutide (200 nmol/L) + high glucose groups was detected by ELISA. *, *P* < 0.05; **, *P* < 0.01; ***, *P* < 0.001

MTT assay results showed that the number of living cells in the high glucose group decreased compared with the blank control and the mannitol treated groups. Compared with the high glucose group, the number of living cells in liraglutide (100 nmol/L) + high glucose, liraglutide (200 nmol/L) + high glucose and liraglutide (500 nmol/L) + high glucose groups increased (Fig. 2A). It was observed that the number of living cells in the liraglutide (200 nmol/L) + high glucose group was the largest, so this group was selected for follow-up experiments. Subsequently, western blot was used to detect the expressions of AMPKα, MAFbx and MuRF1 in blank control, mannitol treated, high glucose and liraglutide (200 nmol/L) + high glucose groups (Fig. 2B-E). The results showed that there were no significant difference in the expression of AMPKα, MAFbx and MuRF1 between the blank control and the mannitol treated groups. Compared with blank control and mannitol treated groups, the expression level of AMPKα in the high glucose group was significantly decreased, while the expression levels of MAFbx and MuRF1 were significantly increased. However, compared with the high glucose group, the expression level of AMPKα in the liraglutide (200 nmol/L) + high glucose group was significantly increased, while the expression levels of MAFbx and MuRF1 were significantly decreased. The ELISA results showed that there were no significant difference in the content of 3-MH in the cell supernatant between the blank control group and the mannitol treated group (Fig. 2F). Compared with blank control and mannitol treated groups, the content of 3-MH in cell supernatant of high glucose group was significantly increased. Compared with the high glucose group, the content of 3-MH in the cell supernatant of the liraglutide (200 nmol/L) + high glucose group was significantly decreased. These results imply that liraglutide can alleviate the decrease of cell viability and degradation of muscle protein caused by high glucose, and improve cell metabolism and mitochondrial activity.

### Identification of DE-mRNAs

**Fig. 3:**
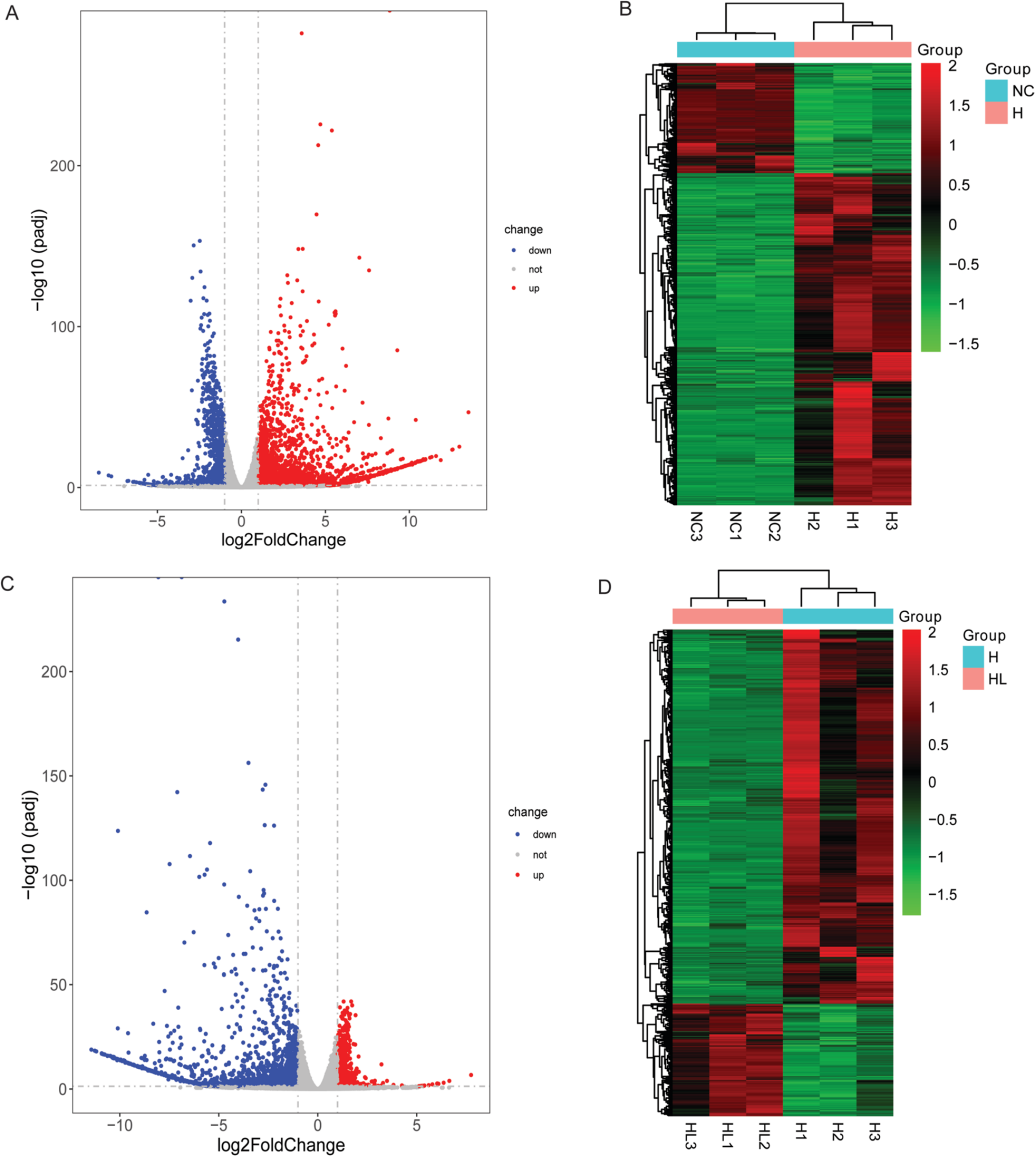
Volcano maps and heat maps of DE-mRNAs in H_VS_NC and HL_VS_H groups. **A**: The volcano map of DE-mRNAs in H_VS_NC; **B**: The heat map of DE-mRNAs in H_VS_NC; **C**: The volcano map of DE-mRNAs in HL_VS_H groups; **D**: The heat map of DE-mRNAs in and HL_VS_H groups

Compared with the NC group, 4594 DE-mRNAs (3450 up-regulated and 1144 down-regulated) were identified in H group. The volcano map and heat map of DE-mRNAs were shown in Fig. 3A and B. The top 10 up/down-regulated DE-mRNAs was shown in Table 1. Compared with the H group, 2555 DE-mRNAs (591 up-regulated and 1964 down-regulated) were identified in HL group. The volcano map and heat map of DE-mRNAs were shown in Fig. 3C and D. The top 10 up/down-regulated DE-mRNAs was shown in Table 2.

**Table 1:** The top 10 up/down-regulated DE-mRNAs in H_VS_NC group

Gene ID	Gene name	Log2FoldChange	*P*-value	Padj	Up/Down
ENSMUSG00000033491.14	Prss35	8.830534581	0	0	Up
ENSMUSG00000092274.4	Neat1	3.590148425	4.63E-287	5.94E-283	Up
ENSMUSG00000001642.19	Akr1b3	4.70198225	2.64E-230	2.25E-226	Up
ENSMUSG00000033327.19	Tnxb	5.386901067	2.33E-226	1.49E-222	Up
ENSMUSG00000026576.13	Atp1b1	4.573804946	3.80E-217	1.95E-213	Up
ENSMUSG00000003545.4	Fosb	4.474193915	4.82E-174	2.06E-170	Up
ENSMUSG00000021792.16	Prxl2a	3.652565139	1.91E-152	5.43E-149	Up
ENSMUSG00000079092.5	Prl2c2	3.391691834	2.67E-152	6.84E-149	Up
ENSMUSG00000055030.2	Sprr2e	7.02260079	6.40E-147	1.49E-143	Up
ENSMUSG00000024621.17	Csf1r	7.601041537	5.76E-139	1.23E-135	Up
ENSMUSG00000020649.12	Rrm2	-2.478556339	1.59E-157	5.82E-154	Down
ENSMUSG00000017499.16	Cdc6	-2.8416497	1.16E-154	3.73E-151	Down
ENSMUSG00000042489.16	Clspn	-2.429980495	3.13E-138	6.17E-135	Down
ENSMUSG00000040204.7	Pclaf	-2.923852259	3.22E-134	5.51E-131	Down
ENSMUSG00000005470.9	Asf1b	-2.198047032	2.36E-128	3.36E-125	Down
ENSMUSG00000028212.17	Ccne2	-2.265144939	1.90E-121	2.43E-118	Down
ENSMUSG00000025574.14	Tk1	-2.068384212	6.31E-120	7.35E-117	Down
ENSMUSG00000001506.11	Col1a1	-3.0212234	7.81E-120	8.71E-117	Down
ENSMUSG00000025001.12	Hells	-1.905389448	1.02E-111	9.05E-109	Down
ENSMUSG00000006678.7	Pola1	-2.219044626	2.21E-111	1.83E-108	Down

**Table 2:** The top 10 up/down-regulated DE-mRNAs in HL_VS_H group

Gene ID	Gene name	log2FoldChange	*P*-value	Padj	Up/Down
ENSMUSG00000022055.8	Nefl	1.688518748	4.56E-45	1.20E-42	Up
ENSMUSG00000001403.14	Ube2c	1.306762846	4.78E-45	1.24E-42	Up
ENSMUSG00000031636.8	Pdlim3	1.740105471	3.39E-43	8.36E-41	Up
ENSMUSG00000023885.10	Thbs2	1.124304594	3.29E-42	7.66E-40	Up
ENSMUSG00000042489.16	Clspn	1.50423827	6.24E-42	1.41E-39	Up
ENSMUSG00000028312.20	Smc2	1.359881852	1.21E-40	2.61E-38	Up
ENSMUSG00000023940.15	Sgo1	1.454202068	2.16E-40	4.55E-38	Up
ENSMUSG00000030867.8	Plk1	1.491444418	6.83E-40	1.40E-37	Up
ENSMUSG00000024590.9	Lmnb1	1.255394169	1.64E-39	3.34E-37	Up
ENSMUSG00000027379.15	Bub1	1.637705726	6.25E-39	1.26E-36	Up
ENSMUSG00000024621.17	Csf1r	-8.053828486	0	0	Down
ENSMUSG00000033491.14	Prss35	-6.874363867	0	0	Down
ENSMUSG00000001642.19	Akr1b3	-4.720005886	3.29E-238	2.68E-234	Down
ENSMUSG00000079092.5	Prl2c2	-4.021360324	8.11E-220	4.95E-216	Down
ENSMUSG00000056457.7	Prl2c3	-3.500826014	1.17E-160	5.72E-157	Down
ENSMUSG00000014813.10	Stc1	-2.643660893	4.15E-150	1.69E-146	Down
ENSMUSG00000032487.9	Ptgs2	-2.780505259	1.07E-147	3.73E-144	Down
ENSMUSG00000055030.2	Sprr2e	-7.09677126	1.97E-146	6.03E-143	Down
ENSMUSG00000005413.9	Hmox1	-2.679551674	1.47E-130	3.98E-127	Down
ENSMUSG00000056220.15	Pla2g4a	-2.207138065	2.75E-130	6.71E-127	Down

### Functional analysis of the common DE-mRNAs of up-regulated DE-mRNAs in H_VS_NC group and down-regulated DE-mRNAs in HL_VS_H group

**Fig. 4:**
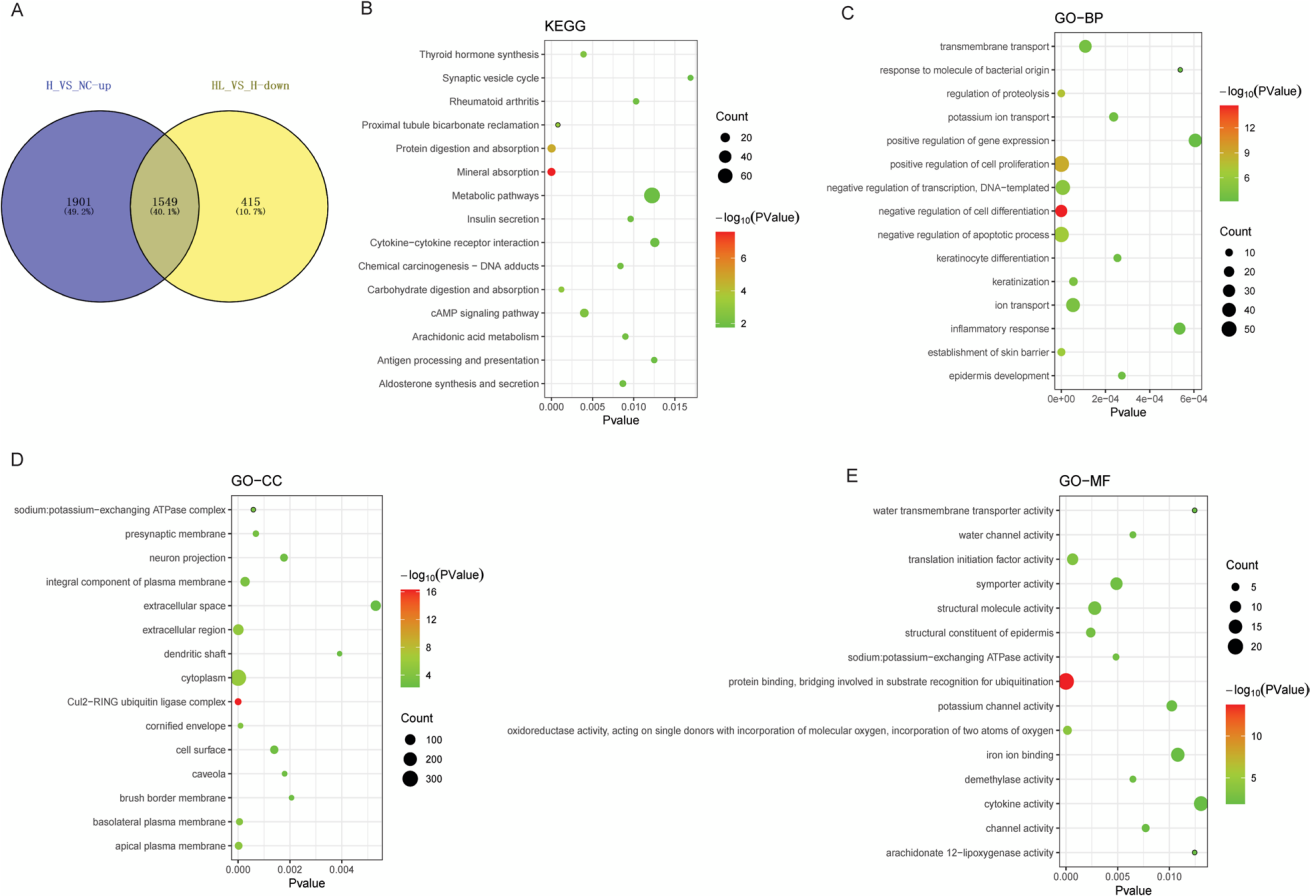
Functional annotation of the common DE-mRNAs of up-regulated DE-mRNAs in H_VS_NC group and down-regulated DE-mRNAs in HL_VS_H group. **A**: Venn diagram of up-regulated DE-mRNAs in H_VS_NC group and down-regulated DE-mRNAs in HL_VS_H group; **B**: Top 15 significantly enriched KEGG terms of the common DE-mRNAs of up-regulated DE-mRNAs in H_VS_NC group and down-regulated DE-mRNAs in HL_VS_H group; **C**: Top 15 significantly enriched GO:BP terms of the common DE-mRNAs of up-regulated DE-mRNAs in H_VS_NC group and down-regulated DE-mRNAs in HL_VS_H group; **D**: Top 15 significantly enriched GO:CC terms of the common DE-mRNAs of up-regulated DE-mRNAs in H_VS_NC group and down-regulated DE-mRNAs in HL_VS_H group; **E**: Top 15 significantly enriched GO:MF terms of the common DE-mRNAs of up-regulated DE-mRNAs in H_VS_NC group and down-regulated DE-mRNAs in HL_VS_H group

The up-regulated DE-mRNAs in the H_VS_NC group and the down-regulated DE-mRNAs in the HL_VS_H group were intersected. A total of 1549 common DE-mRNAs were obtained (Fig. 4A). Subsequently, GO and KEGG analyses of 1549 common DE-mRNAs were performed. KEGG enrichment analysis showed that the common DE-mRNAs were significantly enriched in multiple signaling pathways such as metabolic pathways, cytokine-cytokine receptor interaction, cAMP signaling pathway, protein digestion and absorption and mineral absorption (Fig. 4B). GO enrichment analysis also showed that common DE-mRNAs were significantly enriched in a variety of biological processes, such as positive regulation of cell proliferation, cytoplasm and protein binding, bridging involved in substrate recognition for ubiquitination (Fig. 4C-E).

### Identification of multicentric DE-mRNAs base on the common DE-mRNAs of up-regulated DE-mRNAs in H_VS_NC group and down-regulated DE-mRNAs in HL_VS_H group

**Fig. 5:**
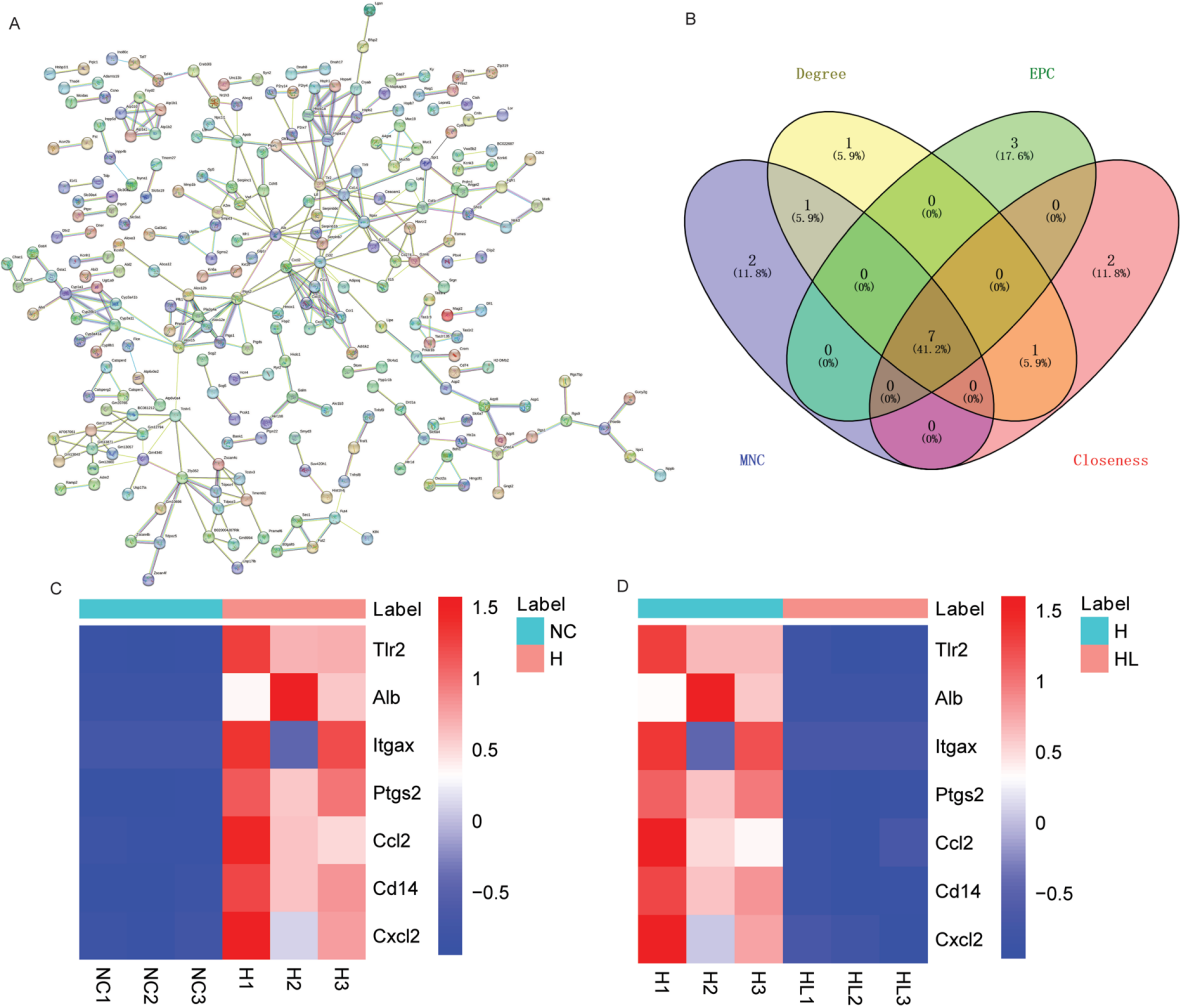
Identification of multicentric DE-mRNAs base on the common DE-mRNAs of up-regulated DE-mRNAs in H_VS_NC group and down-regulated DE-mRNAs in HL_VS_H group. **A**: Construction of PPI network of the common DE-mRNAs of up-regulated DE-mRNAs in H_VS_NC group and down-regulated DE-mRNAs in HL_VS_H group; **B**: 7 multicentric DE-mRNAs were identified using degree, EPC, MNC and Closeness; **C**: Expression heat map of 7 multicentric DE-mRNAs in H_VS_NC group; **D**: Expression heat map of 7 multicentric DE-mRNAs in HL_VS_H group

A PPI network was constructed base on the common DE-mRNAs of up-regulated DE-mRNAs in H_VS_NC group and down-regulated DE-mRNAs in HL_VS_H group. The PPI network includes 321 interaction pairs. The highest interaction score was ATPase, Na + /K + transporting, alpha 1 polypeptide (Atp1a1)-ATPase, Na + /K + transporting, beta 1 polypeptide (Atp1b1), and the interaction score was 0.999 (Fig. 5A). Subsequently, MNC, degree, EPC and closeness algorithms were used to screen multicentric DE-mRNAs based on PPI networks. After the top 10 DE-mRNAs of each algorithm were intersected, a total of 7 multicentric DE-mRNAs were screened out, namely C–C motif chemokine ligand 2 (Ccl2), integrin alpha X (Itgax), toll-like receptor 2 (Tlr2), CD14 antigen (Cd14), C-X-C motif chemokine ligand 2 (Cxcl2), albumin (Alb) and prostaglandin-endoperoxide synthase 2 (Ptgs2) (Fig. 5B). The expression heat maps of these 7 multicentric DE-mRNAs in the H_VS_NC and HL_VS_H groups were shown in Fig. 5C and D.

### Functional analysis of the common DE-mRNAs of down-regulated DE-mRNAs in H_VS_NC group and up-regulated DE-mRNAs in HL_VS_H group

**Fig. 6:**
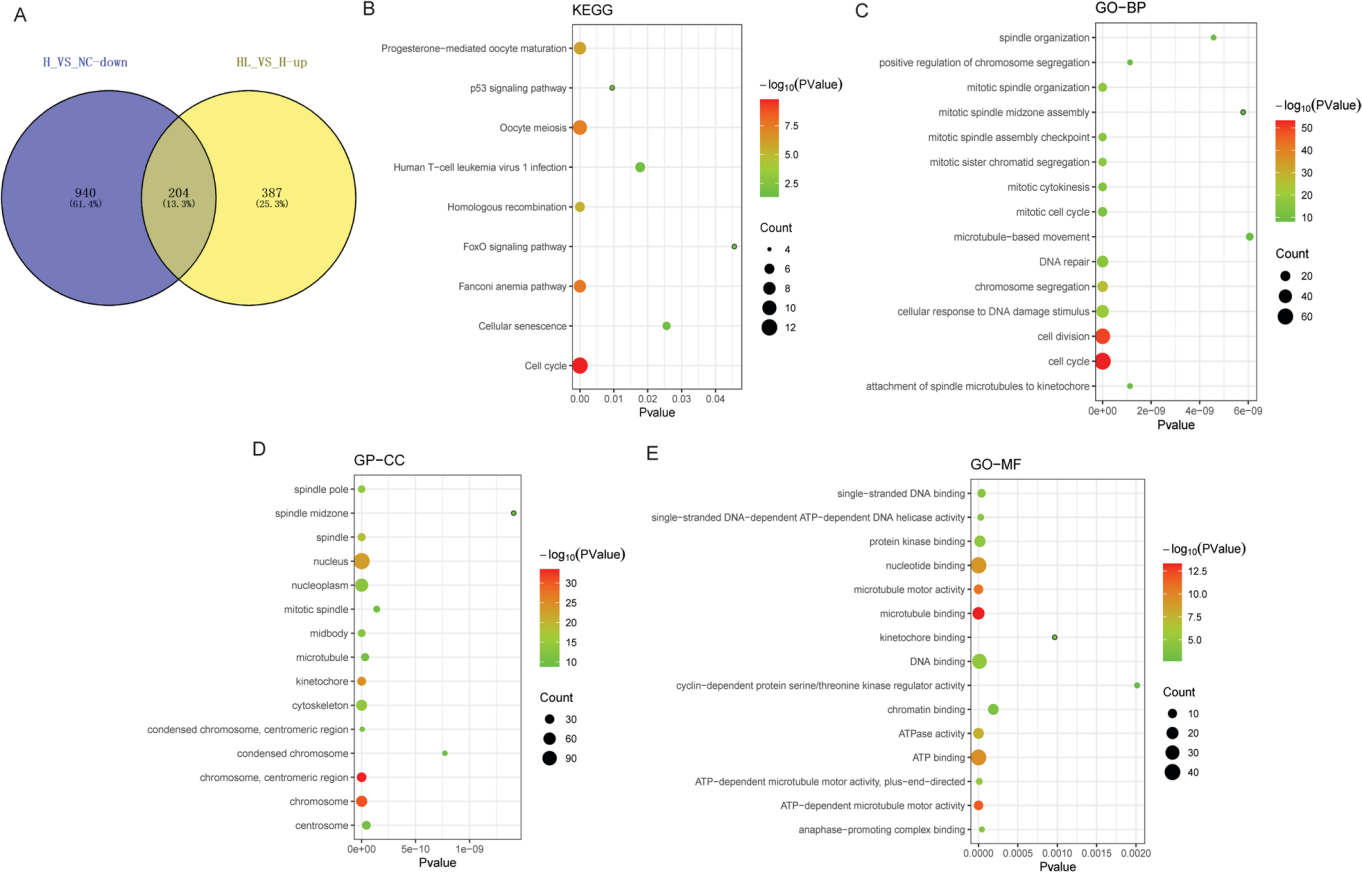
Functional annotation of the common DE-mRNAs of down-regulated DE-mRNAs in H_VS_NC group and up-regulated DE-mRNAs in HL_VS_H group. **A**: Venn diagram of down-regulated DE-mRNAs in H_VS_NC group and up-regulated DE-mRNAs in HL_VS_H group; **B**: Significantly enriched KEGG terms of the common DE-mRNAs of down-regulated DE-mRNAs in H_VS_NC group and up-regulated DE-mRNAs in HL_VS_H group; **C**: Top 15 significantly enriched GO:BP terms of the common DE-mRNAs of down-regulated DE-mRNAs in H_VS_NC group and up-regulated DE-mRNAs in HL_VS_H group; **D**: Top 15 significantly enriched GO:CC terms of the common DE-mRNAs of down-regulated DE-mRNAs in H_VS_NC group and up-regulated DE-mRNAs in HL_VS_H group; **E**: Top 15 significantly enriched GO:MF terms of the common DE-mRNAs of down-regulated DE-mRNAs in H_VS_NC group and up-regulated DE-mRNAs in HL_VS_H group

The down-regulated DE-mRNAs in the H_VS_NC group and the up-regulated DE-mRNAs in the HL_VS_H group were intersected. A total of 204 common DE-mRNAs were obtained (Fig. 6A). Subsequently, GO and KEGG analyses of 204 common DE-mRNAs were performed. KEGG enrichment analysis showed that the common DE-mRNAs were significantly enriched in multiple signaling pathways such as cell cycle, oocyte meiosis, fanconi anemia pathway, progesterone-mediated oocyte maturation and homologous recombination (Fig. 6B). GO enrichment analysis also showed that common DE-mRNAs were significantly enriched in a variety of biological processes, such as cell cycle, nucleus, nucleotide binding and ATP binding (Fig. 6C-E).

### Identification of multicentric DE-mRNAs base on the common DE-mRNAs of down-regulated DE-mRNAs in H_VS_NC group and up-regulated DE-mRNAs in HL_VS_H group

**Fig. 7:**
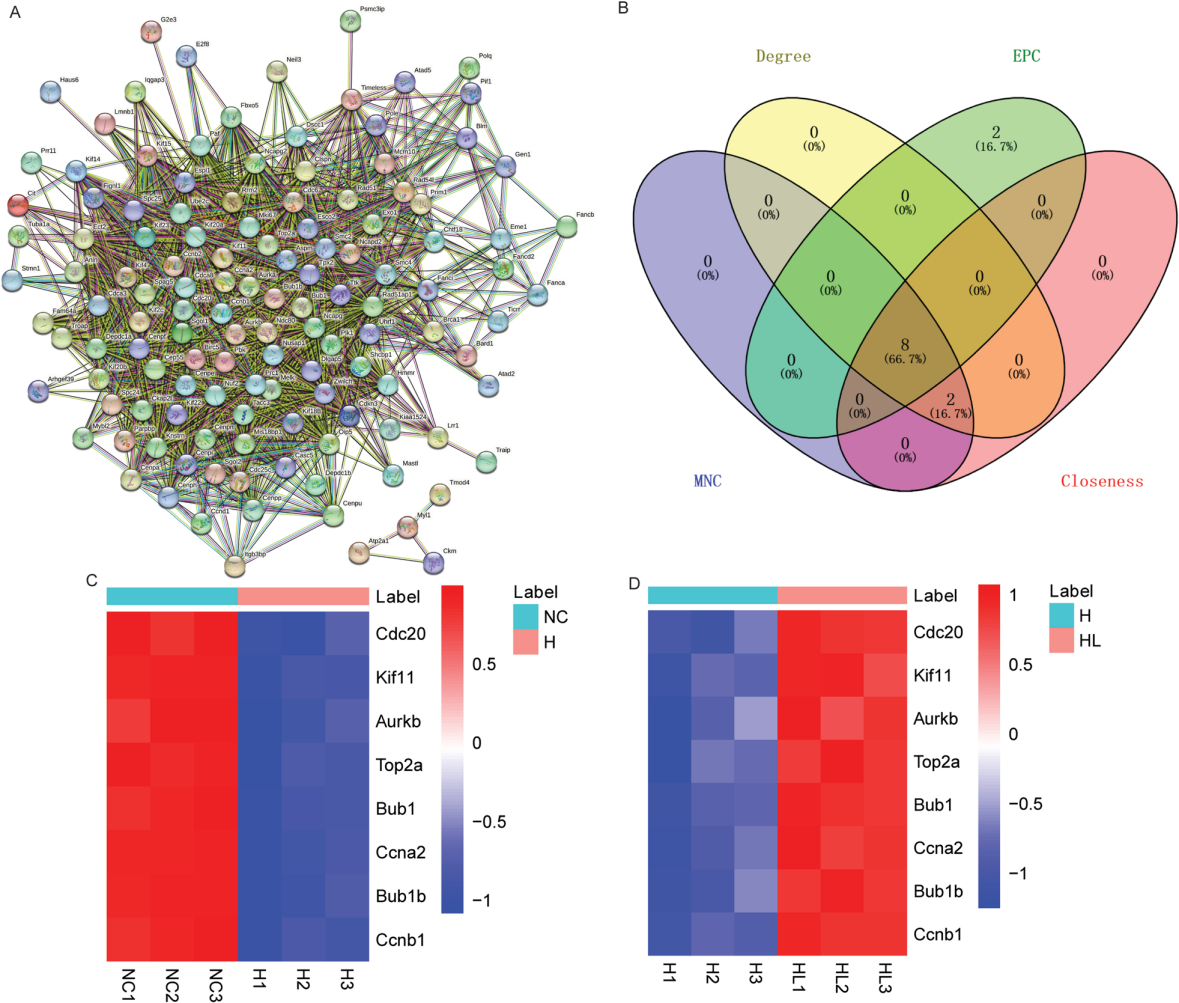
Identification of multicentric DE-mRNAs base on common DE-mRNAs of down-regulated DE-mRNAs in H_VS_NC group and up-regulated DE-mRNAs in HL_VS_H group. **A**: Construction of PPI network of the common DE-mRNAs of down-regulated DE-mRNAs in H_VS_NC group and up-regulated DE-mRNAs in HL_VS_H group; **B**: 8 multicentric DE-mRNAs were identified using degree, EPC, MNC and Closeness; **C**: Expression heat map of 8 multicentric DE-mRNAs in H_VS_NC group; **D**: Expression heat map of 8 multicentric DE-mRNAs in HL_VS_H group

A PPI network was constructed base on the common DE-mRNAs of down-regulated DE-mRNAs in H_VS_NC group and up-regulated DE-mRNAs in HL_VS_H group. The PPI network includes 2769 interaction pairs. The highest interaction score was 0.999 (Fig. 7A). Subsequently, MNC, degree, EPC and closeness algorithms were used to screen multicentric DE-mRNAs based on PPI networks. After the top 10 DE-mRNAs of each algorithm were intersected, a total of 8 multicentric DE-mRNAs were screened out, namely cyclin B1 (Ccnb1), cyclin A2 (Ccna2), BUB1, mitotic checkpoint serine/threonine kinase (Bub1), aurora kinase B (Aurkb), BUB1B, mitotic checkpoint serine/threonine kinas (Bub1b), kinesin family member 11 (Kif11), cell division cycle 20 (Cdc20) and topoisomerase (DNA) II alpha (Top2a) (Fig. 7B). The expression heat maps of these 8 multicentric DE-mRNAs in the H_VS_NC and HL_VS_H groups were shown in Fig. 7C and D.

### AS analysis of H_VS_NC and HL_VS_H groups

**Fig. 8:**
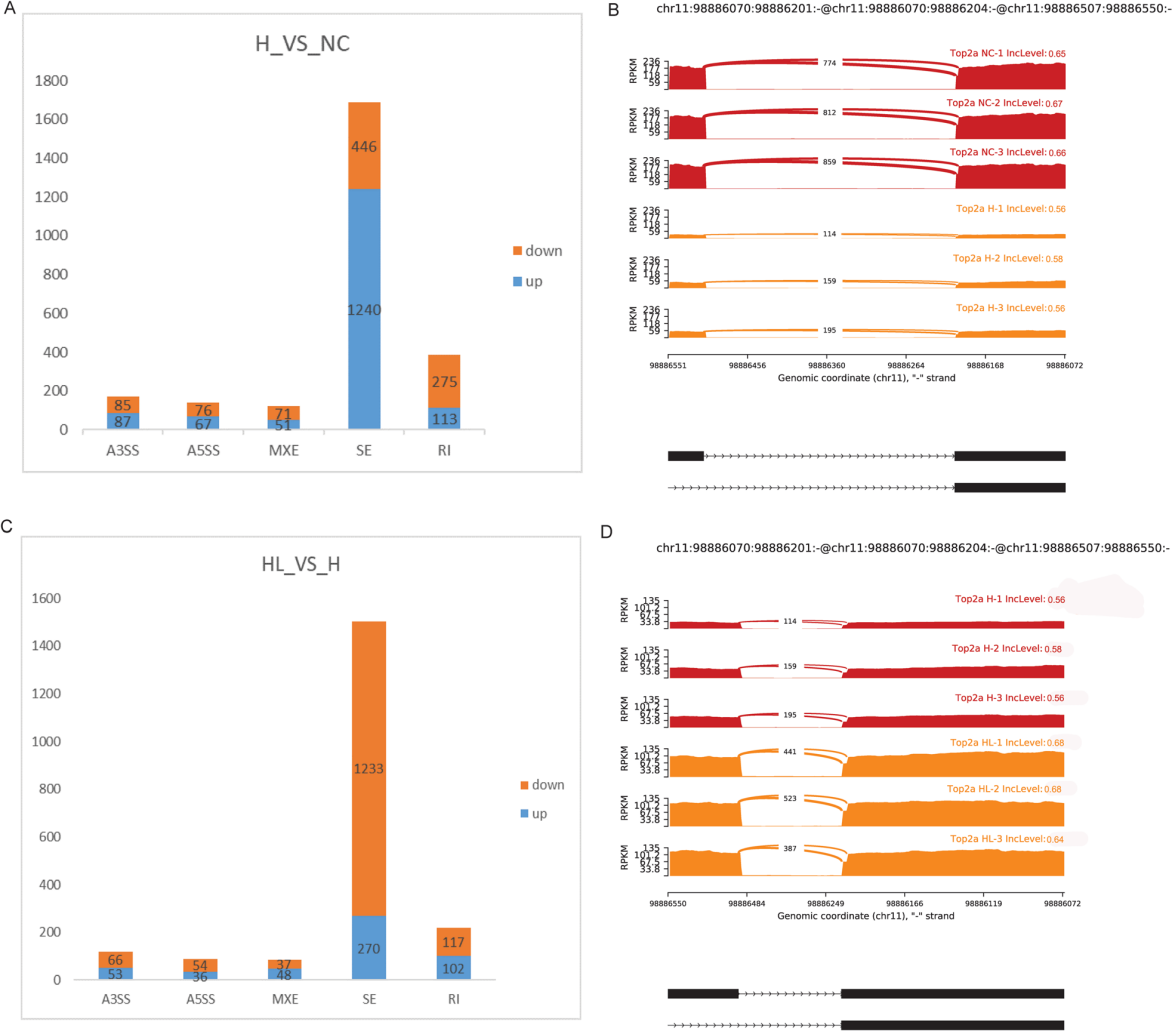
AS difference analysis of H_VS_NC and HL_VS_H groups. **A**: Histogram of AS events with significant differences in the H_VS_NC group; **B**: The detailed sashimi plot for Top2a. Red represents the NC group, and orange represents the H group. **C**: Histogram of AS events with significant differences in the HL_VS_H group; **D**: The detailed sashimi plot for Top2a. Red represents the H group, and orange represents the HL group

The AS difference analysis of H_VS_NC group identified 2511 significantly different AS events, including 172 A3SS (87 up-regulated and 85 down-regulated), 143 A5SS (67 up-regulated and 76 down-regulated), 122 MXE (51 up-regulated and 71 down-regulated), 1686 SE (1240 up-regulated and 446 down-regulated), 388 RI (113 up-regulated and 275 down-regulated) (Fig. 8A). Analysis of 15 multicentric DE-mRNAs showed that Top2a had A3SS type AS in the H_VS_NC group (Fig. 8B). The sashimi plot showed that the exon of Top2a in H group was shorter. The AS difference analysis of HL_VS_H group identified 2016 significantly different AS events, including 119 A3SS (53 up-regulated and 66 down-regulated), 90 A5SS (36 up-regulated and 54 down-regulated), 85 MXE (48 up-regulated and 37 down-regulated), 1503 SE (270 up-regulated and 1233 down-regulated), 219 RI (102 up-regulated and 117 down-regulated) (Fig. 8C). Analysis of 15 multicentric DE-mRNAs showed that Top2a had A3SS type AS in the HL_VS_H group (Fig. 8D). The sashimi plot showed that the exon of Top2a in HL group was longer. These results suggest that Top2a has significant AS event between normal-disease-treatment.

### Expression validation of Ccl2, Kif11, Cdc20 and Top2a by real-time PCR

Ccl2, Kif11, Cdc20 and Top2a were randomly selected from the multicentric DE-mRNAs for real-time PCR validation. All primers for real-time PCR validation are shown in Table S1. Compared with the NC group, the expression level of Ccl2 in group H was increased, while the expression levels of Kif11, Cdc20 and Top2a were decreased (Fig. S1). Compared with the H group, the expression level of Ccl2 in group HL was decreased, while the expression levels of Kif11, Cdc20 and Top2a were increased (Fig. S1). The real-time PCR results were consistent with the above sequencing results.

## Discussion

In this study, effects of liraglutide on myoblasts stimulated by high glucose were investigated. MTT results showed that liraglutide could alleviate the decrease in C2C12 myoblasts viability caused by high glucose. AMPK can detect intracellular energy levels and is responsible for regulating the cell's energy supply system to maintain cell metabolism. It has specific regulation of various aspects of mitochondrial biology and homeostasis [16]. Western blot results showed that AMPKα expression levels were significantly decreased in the high glucose group. In addition, compared with the high glucose group, the expression level of AMPKα in the liraglutide + high glucose group was significantly increased. This result implies that liraglutide can improve the metabolism and mitochondrial activity of C2C12 myoblasts in high glucose environments. MAFbx and MuRF1 are considered to be key regulators of muscle protein degradation [14, 15]. MAFbx and MuRF1 also play important regulatory roles in skeletal muscle atrophy and muscle injury [20]. Moreover, a study showed that liraglutide can alleviate low muscle mass by inhibiting the expression of MAFbx and MuRF1 in patients with diabetic muscle atrophy [21]. Western blot results showed that high glucose promoted the expression of MAFbx and MuRF1 in C2C12 myoblasts, and liraglutide could inhibit this phenomenon. 3-MH is a byproduct of actin and myosin degradation [18], and is also a biological indicator of muscle failure and skeletal muscle toxicity [22, 23]. ELISA results showed that high glucose promoted the expression of 3-MH in C2C12 myoblasts, and liraglutide could inhibit this phenomenon. These results suggest that liraglutide may improve skeletal muscle degradation in high glucose environments.

To further explore the molecular mechanism of liraglutide affecting C2C12 myoblasts stimulated by high glucose, high-throughput transcriptome analyses was performed on the NC, H, and HL groups. Compared with the NC group, 4594 DE-mRNAs were identified in H group. Compared with the H group, 2555 DE-mRNAs were identified in HL group. A total of 15 multicentric DE-mRNAs (Ccl2, Itgax, Tlr2, Cd14, Cxcl2, Alb, Ptgs2, Ccnb1, Ccna2, Bub1, Aurkb, Bub1b, Kif11, Cdc20 and Top2a) were identified based on the Cytoscape-CytoHubba plug-in. In this study, Ccl2, Itgax, Tlr2, Cd14, Cxcl2, Alb and Ptgs2 were common DE-mRNAs of up-regulated DE-mRNAs in H_VS_NC group and down-regulated DE-mRNAs in HL_VS_H group. Ccl2 is required for the proliferation of macrophages in skeletal muscle, and early infiltration of inflammatory macrophages may be associated with early muscle insulin signaling deficiencies [24]. ITGAX, also known as CD11C, is highly expressed in muscle of type 2 diabetes patients [25]. A study has shown that high glucose induction increases TLR2 expression in vascular smooth muscle cells [26]. Moreover, Tlr2 deficiency can reduce skeletal muscle atrophy in mice [27]. Short-term high-fat high-calorie diet increases the expression of CD14 in skeletal muscle of healthy men accompanied by reduced markers of insulin signalling and development of insulin resistance [28]. CXCL2 may be involved in myoblasts proliferation [29]. Logistic regression analysis showed that ALB was correlated with non-traumatic osteoporotic vertebral compression fractures [30]. ALB may also affect the skeletal muscle mass [31]. Ptgs2, also known as COX2, can participate in regulating the proliferation of myoblasts [32, 33]. Herein, Ccnb1, Ccna2, Bub1, Aurkb, Bub1b, Kif11, Cdc20 and Top2a were common DE-mRNAs of down-regulated DE-mRNAs in H_VS_NC group and up-regulated DE-mRNAs in HL_VS_H group. Ccnb1 and CCNA2 are cyclins, which are involved in regulating the proliferation of myoblasts [34, 35]. A previous study suggested that BUB1 may be regulated by miR-30a-3p to play a role in myoblasts development [36]. Bub1b, also known as BUBR1, encodes a mitotic regulatory factor, and mice carrying monoallelic BubR1 mutations are prone to sarcopenia correlating with mTORC1 hyperactivity [37]. Amabile G et al. have found that Aurkb activity is necessary to maintain the differentiation state of mouse myoblasts [38]. KIF11 mediates mitosis and is involved in cell proliferation, and is also associated with bone metastasis in prostate cancer patients [39]. CDC20 is a cell cycle regulator that has been implicated in myoblasts division and muscle repair [40, 41]. Top2a is a ribozyme that regulates and modifies the topological state of DNA during transcription. TOP2A inhibition can damage mitochondrial function and affect embryonic development [42]. In addition, TOP2A is also involved in the regulation of the muscle system [43]. The above studies suggest that 15 multicentric DE-mRNAs may be involved in influencing myoblasts proliferation and skeletal muscle quality. In combination with this study, it is implied that liraglutide may improve myoblasts activity in high glucose environments by regulating the expression of DE-mRNAs.

Functional enrichment analysis revealed that the common DE-mRNAs were significantly enriched in multiple signaling pathways such as metabolic pathways, cytokine-cytokine receptor interaction, cAMP signaling pathway and cell cycle. Metabolic pathways influence myoblasts state and skeletal muscle remodeling, which are necessary for the growth and development of organisms [44, 45]. Cytokine-cytokine receptor interaction may be involved in regulating myoblasts proliferation and differentiation [46, 47]. The cAMP signaling pathway is essential for the terminal differentiation of myoblasts [48]. The cAMP signaling pathway is also involved in the myogenic differentiation of C2C12 cell [49]. Hyperglycemia can induce cell cycle arrest of myoblasts [50]. The proliferation and differentiation of myoblasts is a necessary condition for skeletal muscle regeneration, while cell cycle arrest inhibits myoblasts proliferation [51]. Therefore, it is speculated that the effect of liraglutide on myoblasts in high glucose environments involves a variety of signaling pathways, which is worthy of further study. In addition, a large number of differentially AS events were found in the H_VS_NC and HL_VS_H groups. The AS is the main mechanism controlling gene expression and protein diversity in higher eukaryotes. Previous studies have shown that AS of genes can regulate myoblasts fusion and differentiation [52, 53]. In this study, analysis of 15 multicentre DE-mRNA showed that Top2a had A3SS type AS in H_VS_NC and HL_VS_H groups. These results suggest that Top2a has significant AS event between normal-disease-treatment. Meanwhile, it is further suggested that Top2a plays an important role in liraglutide alleviating the effects of high glucose environment on myoblast.

The results of MTT, western blot and ELISA implied that liraglutide could alleviate the decrease of cell viability and muscle protein degradation caused by high glucose, and improve cell metabolism and mitochondrial activity. Notably, a large number of DE-mRNAs (such as Ccl2, Itgax, Tlr2, Cd14, Cxcl2, Alb, Ptgs2, Ccnb1, Ccna2, Bub1, Aurkb, Bub1b, Kif11, Cdc20 and Top2a) and signaling pathways (such as metabolic pathways, cytokine-cytokine receptor interaction, cAMP signaling pathway and cell cycle) were identified based on high-throughput transcriptome sequencing technique. In addition, AS difference analysis also identified many significantly different AS events in different groups. The results of transcriptome sequencing data analysis suggest that the molecular mechanism of liraglutide to alleviate the effect of high glucose on myoblasts is complex and worthy of further investigation. This study provides a theoretical basis for the clinical effectiveness of liraglutide in the treatment of skeletal muscle lesions in diabetes.

However, it has to be acknowledged that this study also has certain limitations. A large amount of research is needed on the identified DE-mRNAs and signaling pathways to further demonstrate the molecular mechanism of liraglutide to alleviate the effect of high glucose on myoblasts. In addition, the efficacy of liraglutide needs to be verified clinically.

### Supplementary Information

**Additional file 1: Table S1.** All primers used for real time-PCR.** Figure S1.** Expression validation of Ccl2, Kif11, Cdc20 and Top2a by real-time PCR.

**Additional file 2.** Original blot.

## Data Availability

The dataset generated and analyzed during the current study is available in the Gene Expression Omnibus database. The link and accession number are https://www.ncbi.nlm.nih.gov/geo/query/acc.cgi?acc=GSE254268 and GSE254268, respectively.
