# Supplementary material for: Uncovering the potential molecular mechanism of liraglutide to alleviate the effects of high glucose on myoblasts based on high-throughput transcriptome sequencing technique
Source: BMC Genomics. 2024 Feb 8;25:159. doi: 10.1186/s12864-024-10076-w (PMC10851481; doi:10.1186/s12864-024-10076-w)
Supplement: Supplementary file 1 — Additional file 1: Table S1. All primers used for real time-PCR. Figure S1. Expression validation of Ccl2, Kif11, Cdc20 and Top2a by real-time PCR. [file 12864_2024_10076_MOESM1_ESM.docx]

**Table S1 All primers used for real time-PCR**

| **Primer name** | **Primer sequence (5'to3')** |
| --- | --- |
| Mouse GAPDH-F (Internal reference) | 5-AGGCCGGTGCTGAGTATGTC-3 |
| Mouse GAPDH-R (Internal reference) | 5-TGCCTGCTTCACCACCTTCT-3 |
| CCL2-F | 5-AGCCAACTCTCACTGAAGCC-3 |
| CCL2-R | 5-TCTCCAGCCTACTCATTGGGA-3 |
| KIF11-F | 5-GGCTGGTATAATTCCACGCAC-3 |
| KIF11-R | 5-CCGGGGATCATCAAACATCTG-3 |
| CDC20-F | 5-CAGCCTGGAGACTACATATCCT-3 |
| CDC20-R | 5-CGGAGTGACTGGTCATGTTTC-3 |
| TOP2A-F | 5-AACAAAGGGACCCAAAAATGTCT-3 |
| TOP2A-R | 5-TGTGTTCAACAACAGGGATTCC-3 |


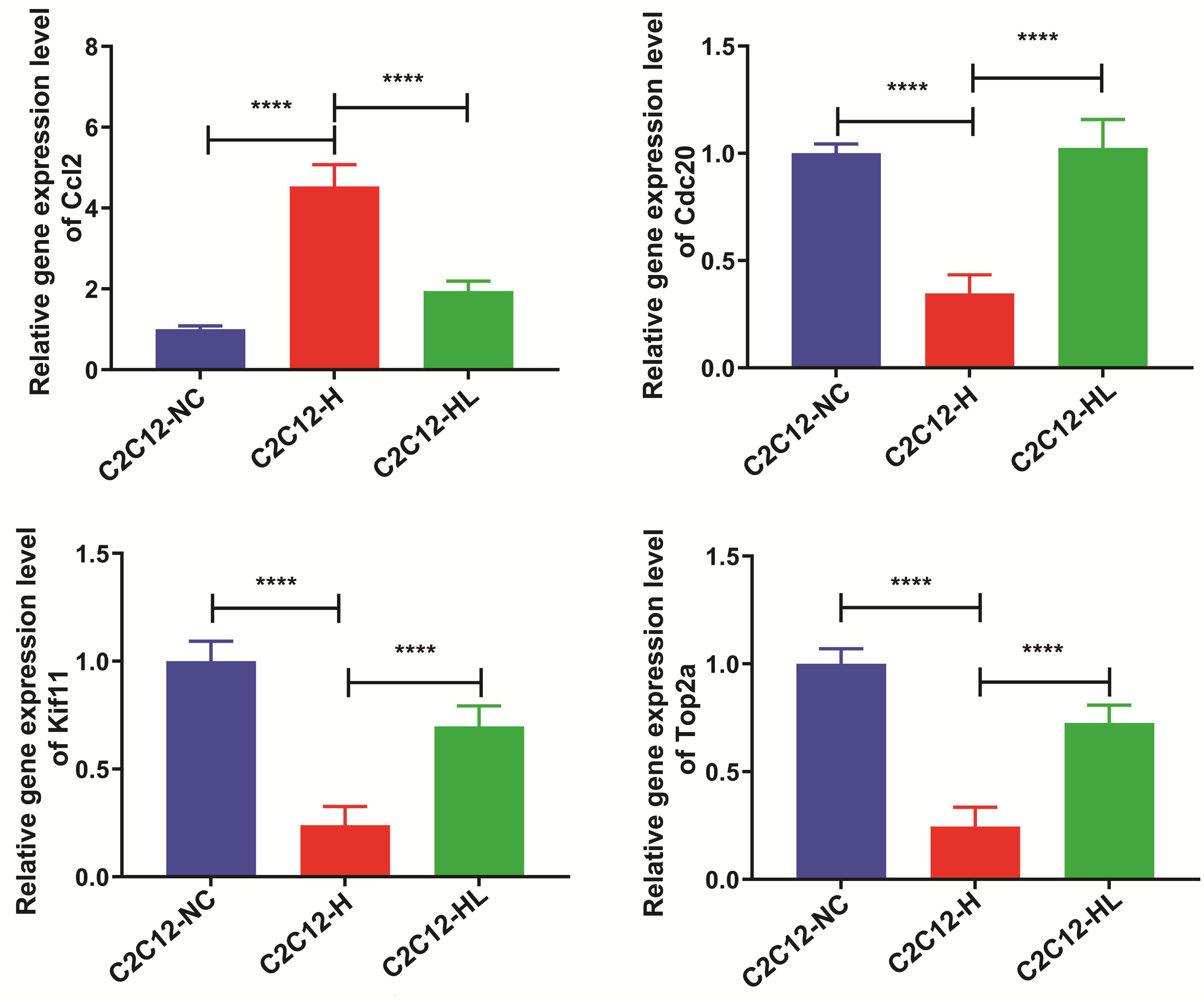


**Figure S1 Expression validation of Ccl2, Kif11, Cdc20 and Top2a by real-time PCR.**
