# Supplementary figures and images for: Uncovering the potential molecular mechanism of liraglutide to alleviate the effects of high glucose on myoblasts based on high-throughput transcriptome sequencing technique
Source: BMC Genomics. 2024 Feb 8;25:159. doi: 10.1186/s12864-024-10076-w (PMC10851481; doi:10.1186/s12864-024-10076-w)

**Original blot**


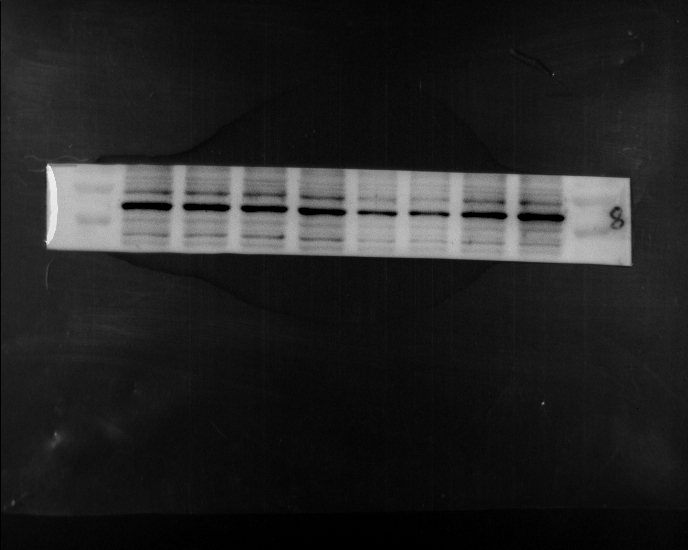


AMPKα


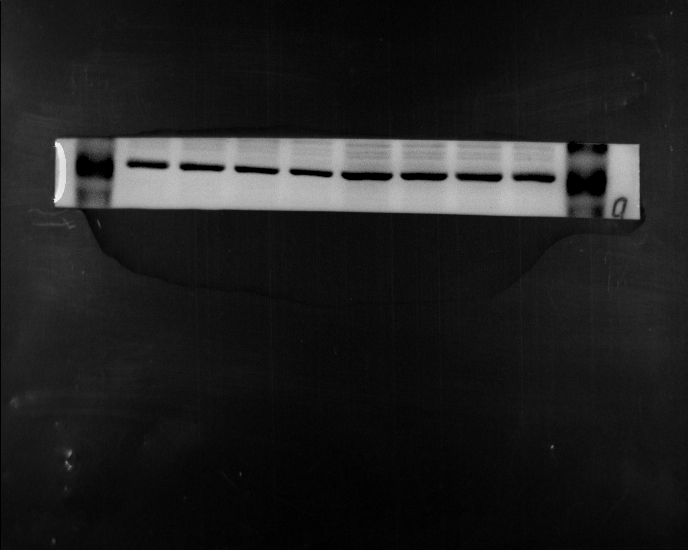


MAFbx


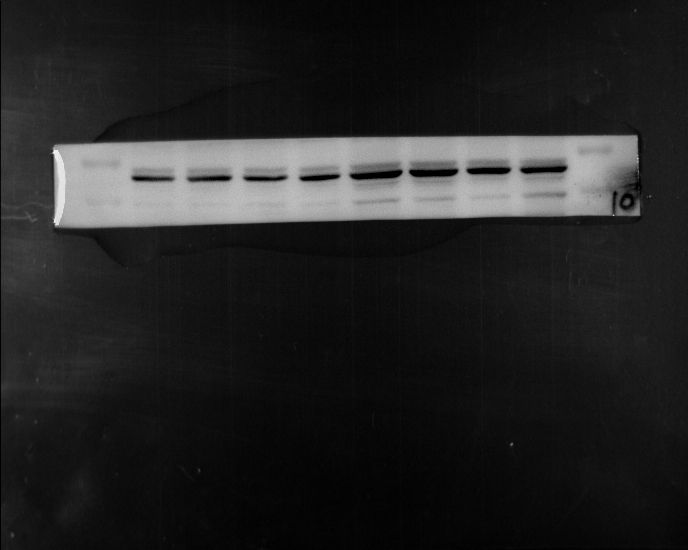


MuRF1


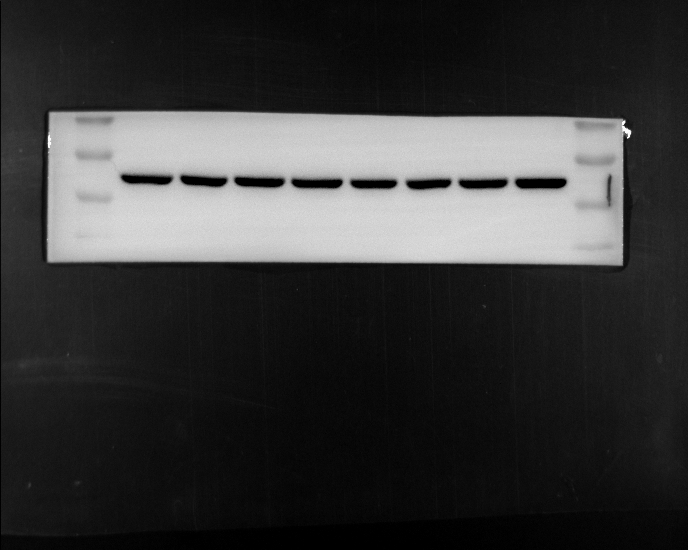


β-actin

Supplement: Supplementary file 2 — Additional file 2. Original blot. [file 12864_2024_10076_MOESM2_ESM.docx]
